# Vitamin D Supplementation for Premenstrual Syndrome-Related inflammation and antioxidant markers in students with vitamin D deficient: a randomized clinical trial

**DOI:** 10.1038/s41598-019-51498-x

**Published:** 2019-10-17

**Authors:** Hajar Heidari, Reza Amani, Awat Feizi, Gholamreza Askari, Shahnaz Kohan, Parastoo Tavasoli

**Affiliations:** 1 Department of Clinical Nutrition, School of Nutrition and Food Science, Food Security Research Center, Isfahan University of Medical Sciences, Isfahan, Iran; 2 Professor of Nutrition. Department of Clinical Nutrition, School of Nutrition and Food Science, Food Security Research Center, Isfahan University of Medical Sciences, Isfahan, Iran; 3 Professor of Biostatistics, Department of Biostatistics and Epidemiology, School of Health, Psychosomatic Research Center, Isfahan University of Medical Sciences, Isfahan, Iran; 4 Associate Professor of Nutrition, Department of Community Nutrition, School of Nutrition and Food Science, Food Security Research Center, Isfahan University of Medical Sciences, Isfahan, Iran; 5 Associate Professor of Reproductive Health, Nursing and Midwifery Care Research Center, School of Nursing and Midwifery, Isfahan University of Medical Sciences, Isfahan, Iran; 6 Molecular Research Lab,, School of Nutrition and Food Sciences, Food Security Research Center, Isfahan University of Medical Sciences, Isfahan, Iran

**Keywords:** Prognostic markers, Nutrition

## Abstract

Premenstrual syndrome (PMS) is a common disorder in the reproductive age that negatively significant impacts on women’s quality of life. This randomized clinical trial study was undertaken to investigate the effect of vitamin D supplementation on inflammatory and antioxidant markers in 44 vitamin D deficient (25(OH)D < 20 ng/mL) students with PMS. Participants received either 50,000 IU vitamin D3 or a placebo pearl fortnightly for 4 months. At the baseline and in the last 2 months of intervention, participants were asked to complete the PMS Daily Symptoms Rating form along with taking the pearls and their blood samples were collected to assess serum levels of 25(OH)D_3_, **Interleukin**10 and 12 (IL-10, IL-12) and total antioxidant capacity (TAC). In vitamin D group, serum levels of IL-10 and IL-12 significantly decreased while TAC significantly increased post-intervention. There were significant differences regarding serum IL-12 and TAC levels between the two groups. Mean score of the total PMS symptoms showed significant improvement in **25(OH)D**. Vitamin D supplementation seems to be an effective strategy to improve inflammation and antioxidant markers in vitamin D deficient women with PMS. This clinical trial was registered at Iranian Registry of Clinical Trials on 20/06/2018 (IRCT20180525039822N1).

## Introduction

Premenstrual syndrome (PMS) is a common disorder in the reproductive age, which includes a wide group of physical, emotional and behavioral symptoms that occur in the late luteal phase of the menstrual cycle and resolving quickly after the onset of menstruation^1,2^. Both physical and psychological symptoms of PMS have significant negative impact on health and quality of life in women^3^. PMS is more common in young women, and according to the WHO, PMS is a public health threat in the modern societies^4^. Around 70–90% of females in their productivity period show at least one of the PMS symptoms. The average prevalence of PMS in the world is 47.8%^5^. In Iranian medical students prevalence of PMS has been reported 39.4% of whom 60.6% were mild, 25.1% moderate and 14.2% severe^6,7^.

While, there is no special physical index or laboratory test for diagnosis of PMS, however, a prospective record of cycle related symptoms is the gold standard for diagnosis^2^. The American Psychiatry Association has published diagnostic criteria for diagnosing PMS^8^.

The exact etiology of this syndrome is unknown, however, some hypotheses exist including; expanded genetic vulnerability, sensitivity to gonadal steroid fluctuations and altered brain processes^5^. None of these hypotheses have yet gained general acceptance, it is theorized that gonadal hormones appear to intervene changes in the activity of central neurotransmitters, such as serotonin and modulation of gamma-aminobutyric acid (GABA) receptors in the brain^9,10^.

PMS has a wide range of stress symptoms including psychiatric and somatic complaints^11^ and oxidative imbalance seems to be involved in the biochemical basis of pathophysiologic mechanisms of PMS^1^. Several studies have shown that luteal phase level of total antioxidant capacity (TAC) is significantly decreased in PMS women^1,5^. Moreover, chronic inflammation, that occurs when cytokine producing cells remain activated, is involved in the etiology of depression and other psychiatric and somatic disorders known as common features of PMS. A cross-sectional study on PMS women revealed that total symptom score was positively associated with IL-2, IL-4, IL-10 and IL-12 and associations were higher for IL-12 and IL-10 suggesting that there are elevated levels of inflammatory factors in PMS^12^.

Although some treatments for PMS are suggested, due to their side effects, high costs and the absence of complete effectiveness of medicines for treatment, most women tend to experience alternative therapies like dietary supplements, minerals and vitamins^3,5^.

It has recently been reported that abnormality of calcium metabolism may be responsible for emotional and physical symptoms of PMS^13^. Regarding the role of vitamin D in calcium absorption and its metabolism regulation, it is proposed that vitamin D deficiency can be associated with PMS^8^.

Vitamin D is a steroid hormone that its biologic actions are mediated through vitamin D receptor (VDR). VDR has been identified not only in calcium-regulating tissues, but also in many other reproductive organs representing a potential role for vitamin D in female reproductive physiology^14^. Moreover, scientific evidence offers that maintenance of an adequate level **of 25(OH)D** is essential in the prevention of a wide variety of health disorders^15^.

Antioxidant role of vitamin D was shown in 1993 through its ability to inhibit iron-dependent liposomal lipid peroxidation. Recently, studies have also recognized the antioxidant properties of this vitamin. Others have proposed other functions of vitamin D such as its anti-inflammatory and immunomodulatory actions^16^.

Researchers have explained vitamin D deficiency (VDD) as a worldwide epidemic^16^. Women with PMS have lower serum levels of vitamin D in the luteal phase compared to their normal controls^8^. Moreover, the highest levels of vitamin D intake (706IU/day) were associated with 41% reduction in PMS vs. the lowest levels (112 IU/day) due to affecting calcium levels, periodic fluctuations in sex hormones and/or modulating neurotransmitters. Therefore, vitamin D deficiency appears to be related to PMS^17^. Cellular studies have demonstrated that vitamin D supplementation decreased the production of inflammatory cytokines and increased anti-inflammatory markers^18^.

Looking at the mechanisms of the effect of vitamin D on PMS and the observed effects of its administration on inflammation and oxidative stress biomarkers which are mediated through Apo-lipoprotein gene expression, parathyroid hormone suppression, repressing NF-kB activation and upregulation of antioxidant systems^19,20^, it is postulated that improving vitamin D status may improve the inflammatory factors and antioxidant capacity and, hence, decrease the incidence and severity of PMS symptoms. According to the best of our knowledge, no study has been conducted in this regard so far. Thus, this study was undertaken to investigate the effect of vitamin D supplementation on inflammation and antioxidant markers in vitamin D deficient (25(OH)D < 20 ng/mL) women with PMS.

## Results

### Baseline characteristics

**Figure 1:**
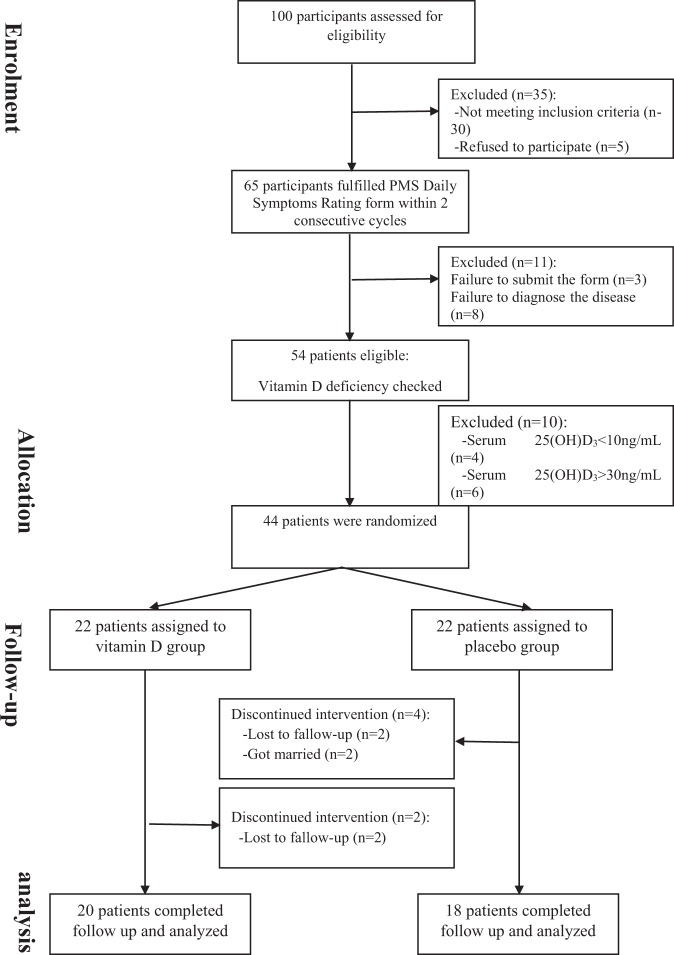
Flowchart of the participants through the study.

During the four months of the study, two participants from the vitamin D group and four participants from the placebo group dropped out of the study (Fig. 1) and data analysis carried out on 38 participants (20 from vitamin D group and 18 from placebo group). Both vitamin D and placebo supplements were well tolerated and no complaints were observed during the study period.

The mean ± SD of students’ age in vitamin D and placebo groups were 21.3 ± 1.6, 21.7 ± 1.8, respectively. As show in Table 1, there were no statistically significant differences between the groups in terms of sociodemographic and obstetrics characteristics as well as the serum 25(OH)D, IL-10, IL-12 and TAC concentrations at the baseline (Table 1).

**Table 1:** Baseline characteristics in the two study groups.

Group	Vitamin D group N = 20	Placebo group N = 18	P-value^a^
Age, years	21.3 ± 1.6	21.7 ± 1.8	0.41
BMI, kg/m^2^	20.7 ± 1.2	**21.4** ± **2.01**	0.21
Waist circumference, Cm	77 ± 8.4	80 ± 10.01	0.59
Menarche age, n (%)			0.51
<12 years	9(40.9)	9(45)	
≥12 years	13(59.1)	11(55)	
Having previous history of PMS (%)	12(54.5)	12(60)	0.48
Habit of adding salt to the table, yes(%)	10(47.6)	9(45)	0.55
Coffee consumption, n (%)			—
≤2 cups/day	22(100)	20(100)	
>2 cups/day	0	0	
Sun exposure (min/day), n (%)			0.78
<30	6(27.3)	8(40)	
30–60	11(50)	9(45)	
60–120	4(18.2)	2(10)	
>120	1(4.5)	1(5)	
Using sunscreen, yes(%)	15(68.2)	17(85)	0.18
Body on sun expose, n (%)			0.20
Only face and hands	18(81.8)	19(95)	
More	4(18.2)	1(5)	
Serum 25(OH)D3, ng/mL	**21.4** ± **7.6**	**21.1** ± **6.8**	0.86
Serum IL-10, Pg/mL	**92** ± **8**	**84** ± **35**	0.49
Serum IL-12, ng/mL	**18** ± **3**	**19** ± **24**	0.89
Serum TAC, U/mL	**13** ± **2**	**19** ± **11**	0.064

^a^Resulted from independent t-test for quantitative and chi-square test for categorical variables Q uantitative variables: mean ± SD Qualitative variables: frequency (percentage).

BMI, body mass index.

Dietary intakes of the participants in each group pre- and post-intervention and between the groups were evaluated (Table 2). No significant differences were seen in dietary intakes of energy, macronutrients and micronutrients between the groups, however, since there were marginal significant differences in energy intake and vitamin E between the two groups, all analyzes were adjusted for the levels of these nutrients further on.

**Table 2:** Dietary intakes of the two study groups.

Nutrients	Vitamin D group N = 20			Placebo group N = 18			P-value^a^
	Baseline	Post-intervention	P-value	Baseline	Post-intervention	P-value	
Energy intake (kcal/day)	**1970** ± **150**	**2010** ± **150**	0.001	**2070** ± **190**	**2110** ± **210**	0.004	0.06
Carbohydrate (g/day)	**170** ± **40**	**170** ± **40**	0.28	**170** ± **50**	**170** ± **50**	0.51	0.60
Protein (g/day)	**48** ± **10**	**47** ± **10**	0.04	**50** ± **13**	**50** ± **10**	0.002	0.30
Fat (g/day)	**45** ± **10**	**44** ± **10**	0.01	**50** ± **20**	**47** ± **20**	0.18	0.87
Linolenic acid (gr)	**0**.**2** ± **0**.**1**	**0**.**3** ± **0**.**12**	0.05	**0**.**2** ± **0**.**1**	**0**.**2** ± **0**.**1**	0.54	0.87
Vitamin A (µg/day)	**410** ± **230**	**400** ± **210**	0.21	**460** ± **270**	**461** ± **260**	0.82	0.70
Vitamin C (mg/day)	**60** ± **19**	**60** ± **18**.**5**	0.11	**67** ± **30**	**60** ± **30**	0.30	0.84
Vitamin E (mg/day)	**12** ± **8**	**12** ± **8**	0.99	**8** ± **7**	**8** ± **6**	0.81	0.04
Vitamin D (µg/day)	**0**.**8** ± **0**.**8**	**0**.**7** ± **0**.**6**	0.10	**1** ± **1**	**1** ± **1**	0.29	0.82
Selenium (µg/day)	**0**.**05** ± **0**.**03**	**0**.**05** ± **0**.**02**	0.85	**0**.**04** ± **0**.**02**	**0**.**04** ± **0**.**02**	0.56	0.29
Dietary fiber (g/day)	**8** ± **2**.**5**	**8**.**1** ± **2**.**6**	0.38	**8** ± **2**	**8** ± **2**	0.81	0.83
Soluble fiber (g/day)	**0**.**4** ± **0**.**2**	**0**.**4** ± **0**.**3**	0.27	**0**.**3** ± **0**.**1**	**0**.**4** ± **0**.**2**	0.09	0.12
Insoluble fiber (g/day)	**2**.**2** ± **0**.**9**	**2**.**3** ± **0**.**75**	0.37	**1**.**9** ± **0**.**1**	**2** ± **0**.**9**	0.02	0.13

Data are shown as mean ± SD.

^a^Resulted from independent t-test.

### Effect of vitamin D supplementation on serum 25(OH)D levels

As described, serum 25(OH)D levels increased by 47% from baseline following supplementation and reached the normal levels (p < 0.001, Table 3).

**Table 3:** Within- and between-group comparison of changes in serum levels of 25(OH)D, inflammatory and antioxidant markers in the two groups of study.

		Vitamin D group N = 20	Placebo group N = 18	P-value^a^	Adjusted P-value^b^
Serum of 25(OH)D (ng/mL)	Baseline	**21** ± **8**	**21** ± **7**	<0.001	<0.001
	Post-intervention	**40** ± **8**	**23** ± **7**		
	Mean difference(%95 CI)	19(14.5,23.4)	2.09(0.12,4.05)		
	P-value	<0.001	0.03		
Serum IL-10 (Pg/mL)	Baseline	**92** ± **8**	**84** ± **35**	0.007	0.017
	Post-intervention	**75** ± **5**	**91** ± **37**		
	Mean difference(%95 CI)	−17.18(−25.4,−8.9)	7.36(−9.4,24.16)		
	P-value	<0.001	0.36		
Serum IL-12 (Pg/mL)	Baseline	**18** ± **3**	**19** ± **24**	<0.001	<0.001
	Post-intervention	**7** ± **1**	**26** ± **24**		
	Mean difference(%95 CI)	−11.3(−17.5,−5.09)	7.04(1.90,12.2)		
	P-value	0.001	0.01		
Serum TAC (U/mL)	Baseline	**13** ± **2**	**19** ± **11**	<0.001	<0.001
	Post-intervention	**21** ± **2**	**16** ± **12**		
	Mean difference(%95 CI)	7.6(4.3,10.9)	−3.3(−6.5,−0.5)		
	P-value	<0.001	0.02		

^a^Resulted from independent samples t-test based on comparing mean difference between two groups.

^b^Resulted from ANOCOVA based on comparing post-intervention values after adjustment for baseline values of outcomes, energy and vitamin E intake.

Data are shown as mean ± SD.

*Significant differences between groups (p < 0.05).

### Effect of vitamin D supplementation on inflammation and antioxidant markers

In vitamin D group, the serum levels of IL-10 and IL-12 decreased by 18.7% and 60.41% after intervention (p < 0.001 and p:0.001, respectively). Moreover, mean serum levels of TAC increased by 36.67% (p < 0.001, Table 3). While, in placebo group, serum IL-12 levels elevated (p = 0.36 and p = 0.01, respectively) and serum TAC levels significantly decreased post-intervention (p = 0.02, Table 3).

### Effect of vitamin D supplementation on mean score of the total PMS symptoms

Mean score of the total PMS symptoms showed significant improvements in vitamin D group (p < 0.001, Table 4).

**Table 4:** Within- and between-group comparison of changes in mean score of the total PMS symptoms in the two study groups.

	Vitamin D group N = 20	Placebo group N = 18	P-value^a^	Adjusted P-value^b^
Baseline	**39** ± **8**	**35** ± **10**	<0.001	<0.001
Post-intervention	**21** ± **6**	**28** ± **8**		
Mean difference(%95 CI)	**−18 (−21**.**5**,**−14**.**8)**	**−7**.**0 (−9**.**5**,**−4**.**5)**		
P-value	<0.001	<0.001		

^a^Resulted from independent samples t-test based on comparing mean difference between two groups.

^b^Resulted from ANOCOVA based on comparing post-intervention values after adjustment for baseline values of outcomes, energy and vitamin E intake.

Data are shown as mean ± SD.

## Discussion

This study was designed to investigate the effect of vitamin D on inflammation and antioxidant markers in vitamin Ddeficient students with PMS in a randomized clinical trial.

The findings indicated that vitamin D supplementation has noticeable effects on both inflammatory markers (IL-10, IL-12) and a significant increase was shown in serum levels of TAC compared to the placebo group. Moreover, improvements in clinical PMS scores were indicated in vitamin D group.

Vitamin D administration fortnightly has been shown to improved serum 25(OH)D concentrations in humans, and none of the treated participants were 25(OH)D deficient (<20 ng/mL) while no adverse or side effects like hypercalcemia were reported^21^. Therefore, we decided to use 50,000 IU vitamin D fortnightly to ensure that there would be no possible interference.

To the best of knowledge, this is the first clinical trial that has investigated the effect of vitamin D supplementation on inflammation and antioxidant markers in PMS.

There are plenty of findings supporting the role for inflammation in PMS. Azizieh *et al*. pointed out to a possible role of pro-inflammatory cytokines IL-8 and TNF-α as a contributing factor in symptoms of PMS^15^. In humans and animals, pro-inflammatory cytokine release results in illness behavior and symptoms of depression, anorexia, anhedonia, paralysis, cognitive dysfunction and reduced social interaction, all of which are common symptoms of PMS. Cytokine expression was shown in the endometrium, ovarian tissue and granulosa cells.

The role of inflammation in PMS was further supported by studies of premenstrual exacerbation of asthma, in which increased levels of airway inflammation during the late luteal phase were associated with worsening of symptoms^12^. CRP, TNF-α and IL-6 have been positively associated with migraine headache, a usual symptom of PMS^22^.

According to Johnson *et al*., there was the strongest correlation between the total score of symptoms and IL-10 and IL-12 levels among inflammatory indices in PMS women^12^.

The correlation analysis between IL-10 and anxiety in Foster *et al*. study showed a positive correlation in the luteal phase in female soccer players with PMS post-game, while this correlation was negative in the group without PMS^9^.

The Mechanism by which vitamin D decreases inflammation remains poorly understood, but based on findings, it seems that the effect of vitamins D on Interleukin is mediated through the action of 1α,25-dihydroxy vitamin D3 that has been reported to inhibit IL-12 production in activated macrophages. Moreover, inhibitory effect of 1α,25(OH)2D3 on IL-12 expression has been presented, while no functional vitamin D response elements (VDREs) has been found in the genes IL-12A or IL-12B^23^. The suppressing effect of 1α,25(OH)2D3 has been associated with the proximal IL-12B promoter, but no VDR direct binding to this area has been found^24^.

This suggests that 1α,25(OH)2D_3_ appears to have double effect on the expression of IL-12B. At the onset of inflammation, the early effect of 1α,25(OH)2D_3_ is cyclical down-regulation of IL-12B expression, until maintaining the immune response on passible level, and later a secondary effect by IL-10 occurs that turns off the IL-12B gene^23,25^.

It is important to note that IL-10, in addition to its anti-inflammatory immune function, has effects on the brain and behavior, taking part in the modulation of mood, anxiety and depression symptoms. Therefore, along with a significant improvement inflammation marker, we observed that mean score of the total PMS symptoms also significantly improved.

There are various arguments on the effect of antioxidants on PMS. Balat *et al*. and Kalia *et al*. assessed the antioxidants levels such as glutathione, ceruloplasmin, SOD and lipid peroxidation product-MDA in PMS. They did not find any significant differences between the control and the PMS women in terms of antioxidant levels^26,27^. On the other hand, some studies reported that TAC levels in luteal phase were decreased in PMS women^1,5^.

Different results seem to be due to the selection of different markers in assessing the effect of antioxidants on PMS and TAC, that includes a set of several antioxidant indices^1^.

Although estrogen is a potent antioxidant, its excessive activity in the luteal phase of PMS women may stimulate its pro-oxidant property leading to reduced TAC levels in these women^27,28^. Therefore, neuronal membrane dysfunction can be secondary to free radical-mediated pathology through toxic effects of free radicals on high ratio of polyunsaturated fatty acid in neuronal membrane, and may affect GABAergic system in PMS women^1^.

Based on our results, increased serum levels of vitamin D after treatment with vitamin D resulted in significant increase in serum TAC levels, reflecting an improvement in antioxidant status in PMS women.

The slight increment in serum vitamin D levels in the controls was possibly due to more sun exposure in the summer, however, controls did not reach the normal range (Table 3). Interestingly, this elevation in vitamin D levels might have led to significant improvement in mean score of the total PMS, again not comparable to the intervention group (Table 4).

Abdollahi *et al*. have indicated that 2,000 IU vitamin D supplementation in vitamin D-deficient young girls with PMS every other day for 12 weeks had no significant impact on PMS symptoms^29^. On the other hand, according to a meta-analysis^3^, vitamin D could exert significant clinical effects on PMS symptoms^30^.

Finally, looking at the possible effects of placebo on premenstrual syndrome, some studies have shown placebo to have significant effects on symptoms of PMS. The response rate to placebo has normally been indicated as 30–40%. It becomes clear that obtaining attention could positively affect the mental status of PMS^31^.

As the limitations of this study, we did not measure serum 1α,25-dihydroxy vitamin D3, calcium and PTH levels to support some aspects of mechanism. We also did not examine the effect of vitamin D on the severity of symptoms and quality of life in women with PMS, which can be new areas for further investigations.

Therefore, results obtained in this study indicate the beneficial effects of 50,000 IU vitamin D3 fortnightly on symptoms and inflammatory and antioxidant markers in vitamin D-deficient students with PMS. Moreover, no adverse effects of supplementation were reported in our participants.

## Methods

### Study design and participants

This study was a randomized, parallel, placebo-controlled, double-blind clinical trial to study the effect of 4 months supplementation with vitamin D (50,000 IU fortnightly) on inflammatory and antioxidant markers and clinical symptoms of premenstrual syndrome in vitamin D deficient university students from December 2017 to August 2018.

Considering the significance level of 0.05 with a statistical power of 80% for detecting at least standardized effect size of Δ = 1 for TAC levels, depression and anxiety scores based on previous studies^8,19^, sample size was determined as 17 participants in each group, an addition of 20% to cover up the possible drop outs. Totally, sample size was considered to be 22 participants in each group.

### Study participants recruitment

At the beginning, 100 female students aged 18–25 years old residing at dormitories of the Isfahan University of Medical Sciences were randomly selected. Participants were included if they had normal body mass index (18.5 ≤ BMI < 25), regular menstrual cycles between 24 to 35 days, being single, not doing exercise professionally, having no evidence of acute or chronic illness and anemia, not using contraceptives, antipsychotics or anti-inflammatory medications and also not using vitamin D supplementation in the last 3 months. Participants were excluded if they got married, were experiencing mental or physical diseases, had serum 25(OH)D levels below 10 ng/mL and not willing to complete the study.

After confirming the participation and filling out the questionnaire regarding the symptoms of PMS, women were assessed for depression and anxiety using Beck Depression Short Inventory (BDI-S) and Beck Anxiety Inventory (BAI), respectively. If a participant was not affected by depression or anxiety disorder, she was asked to fill out the PMS Daily Symptoms Rating form. Sixty-five students were then asked to record their symptoms on a Daily Symptoms Rating form within 2 consecutive menstrual cycles. The questionnaire addressed the most prevalent symptoms of the psychological and physical domains. The participants marked the intensity of their daily symptoms from 0 (not having the symptom), 1 (mild: the symptoms are present, but do not interfere with daily activities such as education and work), 2 (moderate: the symptoms affect the daily activities moderately), or 3 (severe: the symptom prevents the person from taking part in normal daily activities). PMS cases were diagnosed based on the diagnostic criteria of the American Psychiatry Association^8^.

After 2 months, 60 students returned the forms and 54 of them were diagnosed with PMS. Then, a blood sample was taken from each individual to detect the vitamin D deficiency. Finally, 44 students who were simultaneously affected with PMS and vitamin D deficiency (serum 25(OH)D levels 10–30 ng/mL) were selected as subjects and enrolled in the study. The study protocol was approved by the Medical Ethical Committee at Isfahan University of Medical Sciences on 22/01/2017 under grant no. 395734 and ethical code no. IR.MUI.REC.1395,3.734 and Iranian Registry of Clinical Trials (Registration No: IRCT20180525039822N1). All subjects signed the written informed consent to participate in the study.

### Intervention

Participants who were eligible for the study received a randomization number generated by software and randomly divided into intervention and control groups. All participants and researchers were blind to the treatment groups until the statistical analysis was completed. In the intervention group (n = 22), all participants received one oral pearl containing 50,000 IU vitamin D_3_ (D-Vitin 50,000; Zahravi Pharm Co, Tabriz, Iran) and control group (n = 22) received one placebo pearl (Zahravi Pharm Co) every 2 weeks for the 4 months^16,32^. Placebo pearls contained edible paraffin quite identical to the vitamin D_3_ ones in appearance, color, smell, size and package to guarantee the blindness. In the last 2 months of intervention, participants were asked to complete the PMS Daily Symptoms Rating form along with taking the pearls.

### Data collection

A validated semi-quantitative food frequency questionnaire (FFQ) containing 86 items was applied to compare the amount of vitamin D intake before commencing and during the study period (totally 6 months)^33^. The amount of vitamin D received from sunlight was also checked by self-report questionnaire^34^. Anthropometric parameters including height, weight and body mass index (BMI) were measured using standard methods pre- and post-intervention.

### Biochemical analysis

In both groups, blood samples were collected at the baseline and the end of the study in order to assess serum levels of 25(OH)D_3_, Interleukin10 and 12 (IL-10, IL-12) and total antioxidant capacity (TAC). Each time, 5 mL of blood sample was drawn from each participant within one week before the onset of her menstrual period (luteal phase). Serums were collected and stored at −80 °C until further laboratory analyses.

Serum 25(OH)D levels were determined using ELISA kits (bioactiva diagnostica GmbH, Homburg, Germany); according to the manufacturer protocol. Serum concentrations of IL-10, IL-12 and TAC were analyzed using ELISA kits (HANGZHOU EASTBIOPHARM, Torrance, USA) according to the manufacturer protocol in the research laboratory at the School of Nutrition and Food Sciences, Isfahan University of Medical Sciences.

### Statistical analysis

The nutritional status was analyzed by customized Nutritionist IV software. All statistical analyses were carried out using SPSS statistical software version16 (IBM, Chicago, IL, USA). Quantitative variables were presented as mean ± SD while qualitative variables were as frequency (percentage). Normality of quantitative data was evaluated using Kolmogorov–Smirnov test and Q-Q plot. Positive skewed data were subjected to logarithmic transformation. To compare quantitative variables between the groups, independent samples t-test and analysis of covariance (ANCOVA) with adjusting the baseline values of measured outcomes and other confounding variables were used and for categorical variables chi-square test was applied. Within-group comparisons were made using a paired samples t test. The observed effect size for study outcomes were presented as 95% confidence intervals (CI). For all tests, two-sided p-values less than 0.05 were considered statistically significant.

## Ethical approval and informed consent

### Approval

This is to certify that the MSc thesis of Ms. Hajar Heidari that was conducted under the supervision of Dr. Reza Amani had been reviewed by the Research Council of Isfahan University of Medical Science on 22/01/2017 and approved with grant no. 395734 and ethical code no. of IR.MUI.REC.1395,3.734 and registered at Iranian Registry of Clinical Trials on 20/06/2018 (Registration No: IRCT20180525039822N1).

### Accordance

In this study, the methods were carried out in accordance with the confirmed relevant guidelines and regulations.

### Informed consent

All subjects signed the written informed consent to participate in the study.

## References

[1] Duvan CI, Cumaoglu A, Turhan NO, Karasu C, Kafali H (2011). Oxidant/antioxidant status in premenstrual syndrome. Archives of gynecology and obstetrics.

[2] Obeidat BA, Alchalabi HA, Abdul-Razzak KK, Al-Farras MI (2012). Premenstrual symptoms in dysmenorrheic college students: prevalence and relation to vitamin D and parathyroid hormone levels. International journal of environmental research and public health.

[3] Kia AS, Amani R, Cheraghian B (2015). The association between the risk of premenstrual syndrome and vitamin D, calcium, and magnesium status among university students: a case control study. Health promotion perspectives.

[4] Masoumi SZ, Ataollahi M, Oshvandi K (2016). Effect of combined use of calcium and vitamin B6 on premenstrual syndrome symptoms: a randomized clinical trial. Journal of caring sciences.

[5] Fathizadeh S, Amani R, Haghighizadeh MH, Hormozi R (2016). Comparison of serum zinc concentrations and body antioxidant status between young women with premenstrual syndrome and normal controls: A case-control study. International Journal of Reproductive BioMedicine.

[6] Delara M, Borzuei H, Montazeri A (2013). Premenstrual disorders: prevalence and associated factors in a sample of Iranian adolescents. Iranian Red Crescent Medical Journal.

[7] Farrokh-Eslamlou H, Oshnouei S, Heshmatian B, Akbari E (2015). Premenstrual syndrome and quality of life in Iranian medical students. Sexual & Reproductive Healthcare.

[8] Dadkhah H, Ebrahimi E, Fathizadeh N (2016). Evaluating the effects of vitamin D and vitamin E supplement on premenstrual syndrome: A randomized, double-blind, controlled trial. Iranian journal of nursing and midwifery research.

[9] Foster R (2017). Relationship between Anxiety and Interleukin 10 in Female Soccer Players with and Without Premenstrual Syndrome (PMS). Revista Brasileira de Ginecologia e Obstetrícia.

[10] BÄckstrÖm T (2003). Pathogenesis in menstrual cycle‐linked CNS disorders. Annals of the New York Academy of Sciences.

[11] Arranz L, Guayerbas N, De la Fuente M (2007). Impairment of several immune functions in anxious women. Journal of psychosomatic research.

[12] Bertone-Johnson E (2014). Association of inflammation markers with menstrual symptom severity and premenstrual syndrome in young women. Human reproduction.

[13] Shobeiri F, Araste FE, Ebrahimi R, Jenabi E, Nazari M (2017). Effect of calcium on premenstrual syndrome: A double-blind randomized clinical trial. Obstetrics & gynecology science.

[14] Irani M, Merhi Z (2014). Role of vitamin D in ovarian physiology and its implication in reproduction: a systematic review. Fertility and Sterility.

[15] Azizieh FY, Alyahya KO, Dingle K (2017). Association of self-reported symptoms with serum levels of vitamin D and multivariate cytokine profile in healthy women. Journal of inflammation research.

[16] de Medeiros CI (2015). Effect of vitamin D3 supplementation and influence of BsmI polymorphism of the VDR gene of the inflammatory profile and oxidative stress in elderly women with vitamin D insufficiency: Vitamin D3 megadose reduces inflammatory markers. Experimental gerontology.

[17] Bertone-Johnson ER (2005). Calcium and vitamin D intake and risk of incident premenstrual syndrome. Archives of internal medicine.

[18] Hossein-Nezhad A (2013). Evidences of dual role of vitamin D through cellular energy homeostasis and inflammation pathway in risk of cancer in obese subjects. Minerva medica.

[19] Sepehrmanesh Z (2015). Vitamin D Supplementation Affects the Beck Depression Inventory, Insulin Resistance, and Biomarkers of Oxidative Stress in Patients with Major Depressive Disorder: A Randomized, Controlled Clinical Trial, 2. The Journal of nutrition.

[20] Agbalalah T, Hughes SF, Freeborn EJ, Mushtaq S (2017). Impact of vitamin D supplementation on endothelial and inflammatory markers in adults: A systematic review. J Steroid Biochem Mol Biol.

[21] Abbasnezhad A (2016). Effect of vitamin D on gastrointestinal symptoms and health‐related quality of life in irritable bowel syndrome patients: a randomized double‐blind clinical trial. Neurogastroenterology & Motility.

[22] Peterlin B, Bigal M, Tepper S, Urakaze M, Rapoport A (2006). Migraine and adiponectin: is there a connection?. Cephalalgia.

[23] Gynther P (2011). Mechanism of 1α, 25-dihydroxyvitamin D3-dependent repression of interleukin-12B. Biochimica et Biophysica Acta (BBA)-Molecular Cell Research.

[24] D’ambrosio D (1998). Inhibition of IL-12 production by 1, 25-dihydroxyvitamin D3. Involvement of NF-kappaB downregulation in transcriptional repression of the p40 gene. The Journal of clinical investigation.

[25] Matilainen JM (2010). Primary effect of 1α, 25 (OH) 2D3 on IL-10 expression in monocytes is short-term down-regulation. Biochimica et Biophysica Acta (BBA)-Molecular Cell Research.

[26] Kalia G, Sudheendran S, Rao A (2001). Antioxidant status and lipid peroxidation in premenstrual syndrome: a preliminary study. Clinica Chimica Acta 309.

[27] Balat O (2007). Malon dialdehyde, nitrite and adrenomedullin levels in patients with premenstrual syndrome. Archives of gynecology and obstetrics.

[28] Tuladhar ET, Rao A (2010). Plasma protein oxidation and total antioxidant power in premenstrual syndrome. Asian Pacific Journal of Tropical Medicine.

[29] Abdollahi, R., Abiri, B., Sarbakhsh, P., Kashanian, M. & Vafa, M. The Effect of Vitamin D Supplement Consumption on Premenstrual Syndrome in Vitamin D-Deficient Young Girls: A Randomized, Double-Blind, Placebo-Controlled Clinical Trial. *Complementary medicine research*, 1–7, 10.1159/000500016 (2019). 10.1159/000500016 31104056

[30] Arab, A., Golpour-Hamedani, S. & Rafie, N. The Association Between Vitamin D and Premenstrual Syndrome: A Systematic Review and Meta-Analysis of Current Literature. *J Am Coll Nutr*, 1–9, 10.1080/07315724.2019.1566036 (2019) 10.1080/07315724.2019.1566036 31074708

[31] Ebrahimi, E., Motlagh, S. K., Nemati, S. & Tavakoli, Z. Effects of magnesium and vitamin b6 on the severity of premenstrual syndrome symptoms. *Journal of caring sciences* **1**, 183 (2012). 10.5681/jcs.2012.026 PMC4161081 25276694

[32] Sharifi N, Amani R, Hajiani E, Cheraghian B (2014). Does vitamin D improve liver enzymes, oxidative stress, and inflammatory biomarkers in adults with non-alcoholic fatty liver disease? A randomized clinical trial. Endocrine.

[33] Veissi M, Anari R, Amani R, Shahbazian H, Latifi SM (2016). Mediterranean diet and metabolic syndrome prevalence in type 2 diabetes patients in Ahvaz, southwest of Iran. Diabetes & Metabolic Syndrome: Clinical Research & Reviews.

[34] Patwardhan VG (2017). Randomized control trial assessing impact of increased sunlight exposure versus vitamin D supplementation on lipid profile in Indian vitamin D deficient men. Indian journal of endocrinology and metabolism.

